# Online Interventions for the Selective Prevention of Illicit Drug Use in Young Drug Users: Exploratory Study

**DOI:** 10.2196/17688

**Published:** 2020-04-22

**Authors:** Tina Tomazic, Olivera Stanojevic Jerkovic

**Affiliations:** 1 Institute of Media Communications Faculty of Electrical Engineering and Computer Science University of Maribor Maribor Slovenia; 2 Medical Faculty Department of Public Health University of Maribor Maribor Slovenia; 3 National Institute of Public Health Maribor Slovenia

**Keywords:** web-based, intervention, prevention, internet

## Abstract

**Background:**

Digital technologies have a major impact on the daily lives of young people and are also used to seek information on and help with drug-related issues online.

**Objective:**

The aim of this article was to analyze current online interventions for young drug users in Slovenia, with the purpose of contributing to the development of guidelines and key recommendations for effective online interventions.

**Methods:**

This study was part of the project Click for Support. We performed a keyword search, received input from national experts in the field of drug prevention, and conducted an assessment of recognized national online interventions through workshop-based discussions with the target group of 20 young drug users.

**Results:**

The current online intervention services in Slovenia are satisfactory but are still not sufficiently recognized. The most important issues for young drug users were the design and functionality of the online intervention, presence of a clear structure, possibility of using it on smartphones, comprehensive and quick professional feedback, and data security. Playful elements and the ability to share (experiences) with other or former users were also recognized as important.

**Conclusions:**

With effective online interventions, we can include more young drug users, facilitate access to a more affordable service, provide quick professional feedback on patterns of consumption, increase knowledge about the effects and consequences of drugs, and support the reduction or cessation of drug use. From the public health perspective, it is challenging to provide drug interventions broadly to the target group and, hence, decrease inequities.

## Introduction

The growth of social media and increasing popularity of interactive social media platforms [1] have revolutionized methods of communication and influenced the way interactions are handled [2]. Most members of the younger generation are extremely quick to seize opportunities for participation in the new, freely accessible and low-cost model of new technologies [3]. Social networks have also changed with the development of the internet [4]. Furthermore, the increased popularity and capabilities of the internet have led to a revolution in the provision of health-related information and treatment [5].

Public health strategies and policies addressing issues of illicit drug use in Europe are heterogeneous [6]. Traditional public health and clinical interventions, with the central role of health professionals and passive role of patients [7], cannot fully address this need due to resource constraints, limited access to a hard-to-reach population, and other factors. These conditions have spurred innovative approaches to health education and promotion [8]. Because digital technologies such as the internet and smartphone apps have a major impact on the daily lives of young people, there is a greater possibility that young people will search for drug-related assistance online. Approximately 95% of North Americans, 68.9% of the populations of Australia and Oceania, and 85.2% of Europeans use the internet [9]. The United Nations Office on Drugs and Crime describes the use of illicit drugs as a complex health condition that has social, psychological, and biological dimensions and consequences [10].

Online interventions, or web-based interventions (WBIs), are defined as a professional service for selective prevention that is delivered via the internet; includes interactive elements, computer-assisted behavior therapies, education, prevention, and information interventions; and provides individual feedback for young drug users [11,12]. It is possible to deliver interventions to large numbers of people at relatively low cost and ensure that the intervention is accessible 24 hours a day. Therefore, it is available at critical moments, enables anonymity, increases engagement through the use of interactive methods such as video streaming and sharing resources, is economic to run and maintain, and can effectively change health risk behaviors and their determinants [13-15]. Young people with a pattern of drug use can be considered experimental and problematic and are not reached currently. WBIs can offer specialized services in rural areas where distances are too great for easy access to drug treatment centers, which are often concentrated in urban areas. WBIs could also prove to be a cost-effective way of providing support to a larger number of clients than traditional treatment centers, which have limited capacity and human resources [16]. Interventions provided over the internet can overcome traditional barriers to accessing health services [17]. With wide reach and user engagement, social media tools offer a phenomenal opportunity to use social interactions to engage young people in behavior change interventions and to foster socially supportive communities for quitting [18] abuse of illicit substances. These techniques are perceived as reliable, efficient, and able to provide users with useful information and skills, although several aspects require further, in-depth assessment [7]. Online interventions should provide easy access to young people, especially those who would not seek help or advice in a conventional way and cannot be reached through traditional approaches. Another reason why online interventions should be used in day-to-day preventive work is the recent development of new psychoactive substances (NPS), the so-called “legal highs.” According to the European Monitoring Centre for Drugs and Drug Addiction (EMCDDA), there is a general lack of services in the European Union addressing young illicit drug users [11]. As Mounteney et al [19] reported, the purchase of a low threshold over the internet is typical for NPS, as the internet facilitates the movement of products, money, and information across global borders. Social media also plays a role in facilitating the interaction, advertising, and marketing of NPS.

According to Caudevilla [7], WBI also has limitations, as an online forum provides very limited information compared with genuine personal interviews and mediation. Many drug users do not want to enquire about traditional health services because of the possibility of being evaluated or the fear of a professional’s moral prejudices.

There is enough evidence on the effectiveness of online interventions in mental disorders [20-22], harmful alcohol consumption [15,23-34], and smoking [13,18,35-37], whereas the literature is not as cohesive in the field of illicit drugs. Meta-analyses have shown that online mediation has a clear positive effect on the reduction of cannabis use [38-40]. These findings are supported by the evaluation of the Quit the Shit project, which records very positive results in reducing the frequency and quantity of cannabis use [41]. Despite the potential for reaching drug users online, the number of quality intervention choices is still limited, and their effects have not been evaluated sufficiently [42]. In many parts of the world, services designed for drug users simply do not exist.

The aim of this article was to analyze the current online services for young drug users in Slovenia, with the purpose of contributing to the development of guidelines for effective online interventions and to increase the awareness of the importance of online counseling in preventive work with young drug users.

## Methods

The study was a part of the project Click for Support, coordinated by the Landschaftsverband Westfalen-Lippe LWL-Coordination Office for Drug-Related Issues, Münster, Germany, and the National Institute of Public Health of RS as a partner. The project consisted of several phases. First, we reviewed and evaluated online interventions according to predetermined EMCDDA quality criteria, followed by workshops and surveys conducted with young drug users. Finally, key recommendations, or guidelines, for developing effective WBIs were designed. Based on the results of the questionnaire and group discussion and to reach consensus on the final key recommendations, we conducted two rounds [14] of an online Delphi study method to structure the group communication process [43], with the help of 90 external experts. The experts identified strategies and provided recommendations for effective WBIs.

The target group was 20 young people between the ages of 15 and 21 years (average, 17.1 years) with risky consumption of illicit drugs and who had problems with illegal drugs – either previously or at the time of the study [11] (Table 1). Most of the participants smoked cannabis, and some used cocaine, heroin, ecstasy, or amphetamines. Participants were users of the Program Centre for Drug Prevention in Maribor and had been sent to the Program Centre from different institutions including schools and organizations. Some of the participants were long-term Program Centre users, some were just experimenters, and some were sent from schools for a quick intervention.

**Table 1:** Age and gender distribution of the 20 workshop participants.

Characteristic		n (%)
**Age group, years**		
	<14	0 (0)
	14-15	4 (20)
	16-17	8 (40)
	18-21	8 (40)
	>21	0 (0)
**Gender**		
	Male	11 (55)
	Female	9 (45)

To determine the current knowledge of WBIs and their use by young drug users, research was performed using a keyword search (intervention, prevention, offer, new psychoactive drugs, legal highs, illegal drugs, cannabis, cocaine, heroin, app, website, smart phone, internet, social media, chat, forum) and by inquiry among national experts in the field of drugs.

The study was prepared according to the following EMCDDA quality criteria:

1. Interventions should be web-based (eg, websites, apps, or social media app).

2. Interventions should include interactive elements that require the user to do something actively to receive individual feedback.

3. Interventions should be professional services (ie, not simple chats between consumers).

4. The target group should be young drug consumers.

5. The focus should be on illicit drugs, ideally NPS.

6. The effectiveness of the intervention should have been evaluated scientifically.

To show to which extent the criteria were met by the included services, the interventions were ranked according to the number of criteria they fulfilled (“A” for one criterion, “AAAAAA” for all 6 criteria) [11].

The next phase was the assessment of national online interventions with the inclusion of the target group. The first part of the survey addressed the general interest of young people to use WBIs and their prior knowledge and experience with these WBIs, including which elements they had already used. In addition, participants were asked whether anything was missing from the WBIs and which devices they would use to access online services. In the end, the participants were asked to state what features the WBI should have and what kind of information is important. In the section dealing with specific national WBIs, participants were asked whether they wanted to use the app and which specific parts they would use. They were invited to evaluate the attractiveness and usefulness of the service on a scale from 1 to 10, stating which aspects they liked. They were also asked which aspects needed improvement, what they thought the specific service was missing, and, finally, if they would recommend the app to their friends [11]. It is important to gather ideas to motivate users to remain on the website. We suggest incorporating interactive features, such as educational games, fun apps that attract the user to the website, and, of course, consultant feedback. Counselors are expected to have a high level of competence; be kind; give a sense of security, confidence, and motivation; foster a personal connection; and be open. Further, we suggest counseling without a moralizing attitude, but with an attitude that accepts and motivates users. The target group rejects services that promote strict abstinence as the only possible goal, and, consequently, they will not return to such a website.

Participation in the survey and workshop was free and anonymous. The minimum age was 15 years, so we did not request special permission from parents or guardians. Participants were provided a verbal explanation of the course and goal of the research within the project and were provided anonymity. Any data requiring identification of persons could not be included. Participants were offered the opportunity to participate in bowling as an incentive.

## Results

### Results of the Expert Rating of Existing Online Interventions

In Slovenia, 3 websites met the criteria, 1 of which already met the transitional criterion to include expert advice (Table 2).

**Table 2:** Rating of web-based interventions according to the European Monitoring Centre for Drugs and Drug Addiction criteria.

Intervention	Criteria							Rating^g^				
	1^a^	2^b^	3^c^	4^d^	5^e^	6^f^			Online since		URL	
DrogArt	A	A	A	A	A		AAAAA		2006 (Reduser app, 2013)		https://www.drogart.org/	
Med.over.net	A	A	A				AAA		2000		https://med.over.net/	
This is me	A	A	A				AAA		2001		http://www.tosemjaz.net/	

^a^Interventions should be web-based (eg, websites, apps, or social media apps).

^b^Interventions should include interactive elements that require the user to do something actively to receive individual feedback.

^c^Interventions should be professional services (ie, not simple chats between consumers).

^d^The target group should be young drug consumers.

^e^The focus should be on illicit drugs, ideally new psychoactive substances.

^f^The effectiveness of the intervention should have been evaluated scientifically.

^g^A indicates it met one criterion, while AAAAAA means it met 6 criteria.

The website for the DrogArt program includes the associated Reduser app, which represents a rather new approach. It is an interactive tool for self-help that can be used to reduce use of or quit substances. In this app, users record their consumption patterns (consumer logs), feelings, desires, activities, and goals. They can also contact experts for help. With Reduser, providers are striving to help reduce user stress in the situations they face with drugs as well as the stress of their relatives and friends. Expert feedback is available on the DrogArt website via email, apps, forums, Facebook, Skype, and conventional consulting [44]. The target group includes all types of illicit drug and alcohol users, especially high school and university students and people who use club drugs and cocaine.

The website for the Med.Over.Net program answers questions about a healthy lifestyle, exercise, and nutrition; raises awareness on these topics; and offers advice. The website contains information and contacts of experts and institutions that can provide help and advice. Expert feedback is provided through a forum, where experts act as moderators, always encourage users to get help or initiate therapy, and remind them of the effects and consequences of drug use. They also enable communication with medical doctors and professionals [45]. The target group also includes drug users, regardless of their age.

The website for the To sem jaz (“This is me”) community deals not only with the topic of drugs but also all the issues that are important during adolescence, in particular, good self-esteem. Its purpose is to provide youngsters with anonymous, quick, and free access to expert advice from professionals in medicine, psychology, and social work. The website offers information primarily in the form of a forum for questions that are answered by experts. The issues discussed include the side effects of drugs, desires and fears to test substances, feelings under the influence of drugs, and the length of time some medicines may remain in the body or organism [46]. The target group includes adolescents 13-17 years old, especially high school students.

### Reactions to Web-Based Interventions and the Rating of Existing Online Interventions by the Target Group

All 20 adolescents from the target group completed a questionnaire during the workshops. This evaluation of the online interventions revealed that a large percentage of the target group did not use online interventions, although 85% (17/20) were still interested in the use of the selected WBIs. There were positive responses to many parts of the selected WBIs, such as the quality and overall provision of information, design, and possibility of interacting with other users (Table 3).

**Table 3:** Results of the workshop discussion with the target group of adolescents.

Response or issue	DrogArt	Med.over.net	This is me
Number of participants who would like to try the website, n (%)	15 (75)	13 (65)	9 (45)
Number of participants interested in information about drugs, n (%)	18 (90)	17 (85)	16 (80)
Overall attractiveness of the website	Website is attractive	Website is not attractive (advertising is disturbing)	Website is attractive, especially for younger users
Most attractive feature of the website	Set up and structure	Quality of the information	Set up and structure
Effectiveness of the website	Seems to be effective	Does not seem to be effective	Seems to be effective
Number of participants who would recommend it to others, n (%)	15 (75)	8 (40)	7 (35)

The most important issues for the young people in the workshop discussions were the design; a clear structure; functionality, especially the possibility of using the service on smartphones; professional feedback; and data security. Comprehensive and objective information was essential for the target group that accepted the offer. Young people do not want to be lectured, but they need to be encouraged and motivated to change the pattern of drug use. Two of the 3 WBIs sought to incorporate some playful elements, such as quizzes and games, and the ability to share with other (former) users [11].

### Results of the Delphi Study

The 90 international experts included project partners, individuals from the project’s LinkedIn network, and other European experts. They voted anonymously about recommendations for the development and implementation of online interventions that had not yet gained consensus among the project partners. Thus, key recommendations were made regarding the development and implementation of online interventions [11].

Based on the questionnaire responses, feedback from the young people in the discussions, and the Delphi study (see also Jander et al [14] and Neale et al [47]), our key recommendations relate to technical aspects, interactive elements, reaching young drug users, motivation to use the intervention, and evaluation.

For the technical aspects, it is necessary to include experts, clearly lay down provisions, ensure anonymity and data protection in accordance with European Union legislation, and ensure that the setup of mobile versions is user-friendly.

Interactive elements should include entertainment elements, support elements that are as interactive as possible, quizzes, tests, apps, games, blogs, self-tests, animations, and video clips.

To reach young drug users, provide a recognizable offer or unique form; include special elements, access through Facebook, access through YouTube, and personal recommendations; and set up a promotion through the organization’s links and links from other organizations, making sure to include the target group in the promotion.

Provide motivation to use the intervention through the intervention’s structure and usability, content, mode of presentation and attitude, communication between the user and consultant, and transparency.

Make sure to evaluate the effectiveness of the intervention, by planning the process of evaluation from the beginning. Collect data and feedback regularly, and include the target group in the process.

## Discussion

### Principal Findings

Existing online interventions in Slovenia provide enough information to young people about illicit drugs, including the effects and risks of drug use, preventive measures, and professional advice to motivate drug users to reduce their consumption and seek help. However, the results of the questionnaire and feedback from the workshop discussions provide information on young people’s needs and, at the same time, what should be avoided during the development of such interventions to increase their use among young drug users. Various interactive elements, such as self-testing, games, structured intervention programs, quizzes, chat functionality, forums, and email functionality, are recognized as very important [11]. The different participant characteristics in this study that could influence their answers, such as selection of young drug users from the ongoing program at the Centre for Addiction Prevention in Maribor, different statuses of drug use (former and current users), different ages, and the concern of some about being revealed as a drug user must be taken into account. There are practically no drug users that only use NPS; they typically use NPS in combination with traditional drugs, such as cannabis or cocaine. The legal statuses of these drugs are different.

Existing online interventions show significantly positive results in reducing the use of cannabis, tobacco. or alcohol. According to Sindinovic et al [48], over the past decade, treatment providers and policy makers have increasingly recognized the potential of web-based self-help. Therefore, it is recommended that online interventions are also explored for other illicit drug users. It seems that many factors drive change and the development of medicines on the internet. Most of the factors are connected with technology, globalization, and market innovations. Drug markets have become hybrid markets that combine traditional structures of social and economic opportunities with new opportunities offered by the internet [49]. The internet makes it possible to dramatically increase the number of people who can access health care, thereby achieving significant progress in health, among both the population without previous access to health care and general public [50]. Social media can also provide opportunities for creating online communities that support recovery from drug addiction [2].

Given the high level of internet use in Western societies, digital interventions have the potential to reach adolescents everywhere, especially in areas where physical facilities are rare, such as rural communities [16]. Digital interventions can be transmitted and promoted via various channels, such as email-based advertising campaigns aimed at risk groups, including graduates of higher education, military recruits, students in the first year of tertiary education, and unemployed young people in work centers; online advertising via social media, search engines, or music and video sharing sites; mobile advertising via SMS or MMS; mobile apps; and other sources of information (eg, press, television, radio) [39].

Online interventions generally face some criticism for using the internet as a health communication medium. Online communication cannot offer specific and complex forms of personal communication and assistance, which is particularly needed in the therapeutic environment and especially for problematic drug users. Lack of personal contact could increase existing problems because socially isolated people can participate in anonymous internet services potentially without improving their social position. In addition, it is difficult for participants to determine the reliability of a global consultant or online treatment in general.

People with a lack of computer skills or literacy would generally be excluded [9]. The cost-effectiveness of developing online services must be considered. Although apps are likely to be the most successful if they are targeted at a very specific group, the development of technology for small groups is not cost-effective and, therefore, unlikely to be a priority.

Considering the rapid technological process and new developments in the field of illicit drugs, it is very important to remain current in terms of new technologies and content. Funds should be reviewed and updated regularly, to ensure that they remain attractive and meet the needs of modern youth. The development and implementation of effective online interventions require considerable budget, staff, and network resources. In addition, the use of the guidelines depends on staff who have experience with modern technology and social media. A minimum of experience and, more importantly, a willingness to use these technologies are essential when planning to use a WBI [11]. Given these trends, it is not surprising that there has been significant interest in Europe devoted to the development and implementation of interventions and digital solutions delivered through computer and mobile technologies within European health care systems [51]. However, when we talk about reducing the use of illicit drugs, we cannot overlook the fact that it is necessary to reduce a number of factors that lead to drug use, such as poverty, lack of education, unemployment, poor parenting, and bad skills [52]. By understanding why individuals choose to consume the drugs, within the social and cultural context, we can begin to engage in truly helpful conversations about how to reduce drug-related harm [53]. Furthermore, governments should play an active role in strengthening the prevention of illicit drug use through the enforcement of legislative measures to deter access to illicit drugs; inclusion of illicit drug and health education in the curricula of secondary schools, higher secondary schools, and universities; financing of the evaluation of prevention programs and dissemination of piloted programs at the national level; promotion of community programs; and promotion of the exchange of good practices, guidelines, and quality standards at national and international levels. Long-term strategies should be implemented based on cooperation between government institutions, academic institutions, non-governmental organizations, and mass media [54].

### Conclusions

With effective online methods, we could reach a large number of young drug users, facilitate access to more affordable services, provide a quick response or professional feedback on patterns of consumption, increase knowledge about the effects of drugs and their consequences, and support reduction or cessation of drug use. The guidelines, developed as a product of the results of our survey (and surveys from other partners), could improve and increase the effectiveness of existing online services or enable the development of new services designed especially for drug users.

It seems likely that WBIs will continue to develop and that their importance will increase. The speed at which the internet permits the transformation of the drug market will continue to be the main challenge for law enforcement and public health research and monitoring [55]. From the public health perspective, it is challenging to provide such interventions more broadly to the target group and, hence, decrease inequities.

### Future Directions

Although technologies and platforms for accessing interventions via smartphones and tablets are evolving rapidly, the anonymity and protection of data related to the transmission of drug use data on the internet remain a real concern [56]. These issues should be addressed fully in the further development of these measures, because these measures are an excellent basis for developing practical and relevant guidelines for implementing effective online interventions for selective prevention.

Furthermore, based on these findings, there is a recognized need to extend the research regarding the effectiveness of WBI implementation in daily prevention work.

## Abbreviations

EMCDDA: European Monitoring Centre for Drugs and Drug Addiction.
NPS: new psychoactive substances.
WBI: Web-based intervention.
